# The Hospital. Nursing Section

**Published:** 1905-02-11

**Authors:** 


### The Hospital.

### Nursing Section.

Contributions for this Section of "The Hospital" should be addressed to the Editor, "The Hospital," Nursing Section, 28 & 29 Southampton Street, Strand, London, W.C.

No. 959.—Vol. XXXVII. *SATURDAY, FEBRUARY* 11, 1905.

### Notes on News from the Nursing World.

### PRINCESS VICTORIA AND HER NURSES.

Happily, the work of the nurses in attendance on Princess Victoria has not been of a very arduous character. On Saturday it was officially announced that her Royal Highness had made such favourable progress that no further bulletin would be issued. The nurses—Nurse Fletcher, who, with Miss Haines nursed his Majesty at the time of his operation, and Nurse Isaacs, both from Miss McCall's home in Welbeck Street—of course, remain at Buckingham Palace until the patient has quite recovered.

### THE SYSTEM OF WET-NURSING IN RUSSIA.

The remarkable article on "Infantile Mortality in Russia," which appears in another page, is from the pen of a nurse who spent several years in Russia and owing to the position she occupied, had unusual opportunities of becoming acquainted with the subject upon which she writes. Her account of the system of wet-nursing in the Muscovite Empire will be read with great interest and great pain by nurses in this country. The system appears to be about as bad as it could be, and we are not surprised that the head of a maternity institute last year visited over 200 villages searching for likely young women, seeing that in consequence of the appalling ignorance of the means of bringing up babies by hand, the mother who becomes a wet-nurse practically murders her own child. The Massacre of the Innocents which under these circumstances goes on in Russia, is infinitely worse than the massacre of grown-up people who can, at any rate, do something to defend themselves. We cannot for a moment believe that either the humane Tsar, or his gracious Consort, is in the least aware of a state of affairs which in these days ought to be absolutely impossible in any civilised community.

### QUEEN'S NURSES AND THE HOLT-OCKLEY SYSTEM.

Some very definite statements put forward by the promoters of the Holt-Ockley system of nursing in reference to the working and arrangements of Queen's nurses under Queen Victoria's Jubilee Institute have been categorically dealt with by Miss Guthrie Wright, the honorary secretary of the Guthrie branch of the institute. Thus, the allegation that the Queen's nurses are not suitable for country and scattered districts, is met by Miss Wright with the answer that, valuable as Queen's nurses are in town, the advantage of trained district nursing is still greater in country districts, as, the further from the doctor, the more need has there been found of the full training by which the nurse learns not only what to do, but what to leave undone. The statement that in the country difficulties arise because of the working of the district from a central home is disposed of by Miss Wright's rejoinder that no Queen's nurse works in a country district from a central home, each nurse living in the district in which she nurses, under the control of a local committee, with inspection from the training centre. Another allegation is that the rules of the institute debar Queen's nurses from staying over-night; whereas, as Miss Wright shows, the rules allow them to be employed on night duty under exceptional circumstances, and, as a matter of fact, a nurse frequently remains nights, and even weeks, with a critical case. In reply to the assertion that the poor and working classes do not care to avail themselves of the services of highly-trained and educated nurses, Miss Wright has no hesitation in affirming that experience proves the contrary; and as to the contention that when the mother is laid aside by illness Queen's nurses are generally reluctant to undertake the mother's ordinary duties, she says that in any emergency the Queen's nurse shares or does much towards putting the patient's room into nursing order, that she cooks for the patient, and often assists the family in other ways. There remains the plea that persons employed under the Holt-Ockley system, being part general servant and part attendant on the patients are cheaper than Queen's nurses to the committees, who provide and partly support them. On this point the rejoinder of Miss Wright is that they are dearer to the individual patients who have to house, feed, and partly pay for them. We think it is a pity that an attempt to contrast the Holt-Ockley system to the detriment of the system under Queen's nurses work should have been made. There is scope for both under present conditions, but as a comparison has been challenged, Miss Wright cannot be blamed for taking up the cudgels in defence of the institute.

### EXPECTED LOCAL GOVERNMENT BOARD INQUIRY AT PLYMOUTH.

The Plymouth Guardians have adopted the recommendation of the Hospital Committee to ask the Local Government Board for permission to dismiss the superintendent nurse. The Governor requested that the report should not be debated, and the guardian who moved its adoption expressed the regret with which the recommendation was made, and said that when the superintendent nurse came before the committee she was told the reasons why the board wished to receive her resignation. Another guardian asked if the statement made by Miss Holliday with regard to the terms of service of the nurses was a correct one. The Governor replied that the board had not looked into it It was very difficult to go back over these things. Several guardians said that they saw no difficulty whatever, but in the end the recommendation was carried by

18 votes to three. The Local Government Board will doubtless hold an inquiry at Plymouth, and their inspector will certainly look into things, which the board seem to find a task beyond their power.

### THE NURSING SITUATION IN AMERICA.

A PROMINENT American physician, who keeps constantly employed a number of trained nurses, thinks that the difficulty in securing domestic help in the United States is on the increase, and is bound to influence the nursing situation. He says that many families residing in flats or small houses, who are abundantly able to pay servants have none because they can get none. They go out for dinner to a restaurant, and the lady of the house herself prepares the two light meals. Laundry work is sent out of the house, and occasionally a woman is secured by the day to do the heavier cleaning. When the lady of the house is taken ill the man can go out for his meals, but it then becomes a question who will cook for the nurse and patient, if a trained nurse is called in. The physician confesses that, "because trained nurses as a rule object to such service, and help is not to be had, he himself frequently prefers the less skilful woman who will go into the house and meet the emergency genially and resourcefully." Some of his patients, he adds, are glad to pay an untrained woman the same rate they would pay a trained nurse simply because the former handles the domestic situation better. Therefore, though he naturally would rather employ a trained nurse so far as nursing goes, he has often to yield to the wishes of the patient who is better satisfied to know that the home is being cared for than to receive constant attention herself. A similar state of things does not yet prevail on this side of the Atlantic, but there are many signs that it is approaching, and the failure of a full supply of domestic servants here might similarly affect our system of private nursing.

### AN ASYLUM NURSE TRIED FOR KILLING A PATIENT.

AT the Berkshire Assizes on Monday, Edith May Hall, a nurse at Berkshire County Asylum, was indicted for having feloniously killed Gertrude Elizabeth Harris, an inmate of that institution. The case was heard before the Lord Chief Justice, and in the course of the trial one of the witnesses for the prosecution, a nurse, stated that the accused took the deceased by her hair and struck her. But the witness also stated that the woman had struck the accused and other nurses in her frenzy. Medical evidence was to the effect that the deceased suffered from acute mania, and died of hæmorrhage of the brain. The proceedings came to an abrupt close in consequence of the Lord Chief Justice stopping the case with the remark that it should not have been brought before him. The accused was then formally acquitted. The salient facts of this unfortunate business were given in our issue of December 31st, when we reported that the coroner's jury returned a verdict that the patient died from hæmorrhage of the brain caused by excitement while being removed to the lavatory. Referring in our issue of January 14th to the course pursued by the county justices at Wallingford who, in spite of this verdict, committed the nurse on a charge of manslaughter, we said that in face of the statement then made by the medical superintendent of the asylum, that the cause of the hæmorrhage was a blow, we did not see how the magistrates could do otherwise than send the case for trial; and we still think so. But we rejoice that the nurse has been acquitted of the serious crime imputed to her. She has had a very severe lesson, and we hope that it will be a warning to all concerned. The authorities of Moulsford County Asylum are not blameless in the matter. They should not have allowed a young girl to remain on duty in the refractory ward for more than six months. We hope that Nurse Hall will be given a fresh start under more satisfactory conditions.

### A COUNTY ASSOCIATION FOR GLOUCESTERSHIRE.

LAST week an important meeting was held at Gloucester in order to inaugurate the Gloucestershire County Nursing Association. The object, as described in a resolution which was adopted, is to aid and encourage the formation of local nursing societies, and to promote the training of suitable women as nurses and midwives. Sir Michael Hicks-Beach, M.P., who was one of the speakers, said that an annual income of between £400 and £500 would be required, and donations to the amount of £100 were announced.

### DEARTH OF REGISTERED MIDWIVES IN LINCOLNSHIRE.

THE Lincolnshire Nursing Association is an important and admirable organisation, and we are glad to learn that the managers are quite willing to exert themselves in order to remedy as far as possible the dearth of registered midwives which at present prevails in the county. For example, there were formerly 91 midwives known to be acting in the Lindsey division alone, while up to a few days ago the number registered in the division was only seven. This indicates an urgent need, and Lady Thorold, of Syston Park, Grantham, with other generous supporters of the Lincolnshire Nursing Association who are interesting themselves in order to increase the funds so as to secure a sufficient number of qualified and registered midwives are pursuing a course which might with advantage be emulated in other quarters. No doubt the question of co-operation with the Rural Midwives Association has engaged the attention of Lady Thorold and her colleagues.

### THE MIDWIVES ACT AND POOR-LAW INFIRMARIES.

IT was stated at the last meeting of the Aston Guardians that before the passing of the Midwives Act many of the probationers trained in Aston Infirmary obtained certificates from the London Obstetrical Society, but that it would not be possible for them to obtain certificates from the Central Midwives Board. This is, of course, correct, unless or until the Aston Infirmary is recognised by the Board as a training school. The Guardians have, however, made application for recognition, and in support of the application they have expressed themselves confident that four or five probationers yearly could pass the examination.

### A COMPLIMENT TO A SUPERINTENDENT NURSE.

IN expressing his regret at the resignation of the superintendent nurse at Tonbridge Infirmary one of the Tonbridge guardians said he wished that they could have had the opportunity of discussing the question of Miss Tisdell's salary before she accepted another appointment. As superintendent nurse at Farnham Infirmary she is to receive £15 more per annum than at Tonbridge. We do not see, however, how the opportunity could have been given. Perhaps at the end of five years of service she may have supposed that an increase would have been offered her spontaneously. At any rate, the Tonbridge guardians were unanimous in expressing appreciation of her work and regret at her departure. The wards, it was stated, have been much brighter under her care, and the general arrangements have been greatly improved.

### THE VALUE OF A LADIES' GUILD.

From every point of view the proceedings at the fourth annual meeting of the Bradford and District Nursing Association were satisfactory. The number of cases nursed in the year was 1,154, and the number of visits paid was 39,545. To the home patients of the Bradford Royal Infirmary 13,324 visits were paid, these figures showing an increase of 54 patients and 2,151 visits over the previous year. The receipts came to £1,072, including £396 obtained by the Ladies' Guild. This amount is just £200 more than the subscriptions, and shows the exceeding value of having such an organisation as the Ladies' Guild at work, though they did not collect quite so much money as in 1903. The expenses of the association were £992, and the balance in hand is £80, as compared with £49 at the beginning of 1904. Judging by the enthusiasm prevailing at the meeting, we do not doubt that the need for augmenting the nursing staff will be recognised by the people of Bradford, who would not find it very difficult to increase the income to £1,200.

### PRESENTATION OF CERTIFICATES.

We understand that seven nurses at Lewisham Workhouse Infirmary, having passed their examinations, have just been presented by the Chairman of the Board of Guardians with their certificates. In the course of his remarks, he said that the system of training at the infirmary had been successful for many years, and the Guardians all hoped that the future career of those who had now received certificates would be as satisfactory in nursing as was promised by their efforts as probationers. The successful nurses are Miss Edith E. Hope, Miss Ethel L. Willoughby, Miss Annie Ellis, Miss Annie Angell, Miss Harriet I. Hambrook, Miss E. Dellar Ford, and Miss Effie May Allen.

### TWELVE MONTHS OF DISTRICT MATERNITY WORK.

An interesting report of twelve months' work by a district nurse in the villages of Durweston, Bryanston, Stourpaine, Pimperne, Blandford St. Mary, and Langton, in Dorsetshire, has been sent to us. Last year was her second year, and the number of visits increased from 770 to 1,252. The nurse lives at Blandford, and the villages are all adjoining. Except in bad weather, after dark, urgent cases, or heavy pressure of work, she uses her own bicycle. When a conveyance is necessary, it is paid for out of the funds. There is no committee, and Mrs. Heber Percy, whose father, Lord Portman, subscribes largely to the fund, and supplies wine, brandy, and tickets for convalescent homes to the patients, makes her own rules, examines the nurse's report, and signs it. There is a referee in each village who gives tickets to all the poor people. The very poor are attended free of all charge. A wise precaution exercised by the nurse is to send any not over clean linen to be properly disinfected before it is returned to her. The balance-sheet, which has been duly audited, shows that the receipts for the year were £127 12s. 10d., including £14 14s. 4d. for patients' fees, and the expenses £25 8s. 3d. less. The fees are very moderate, the rules are simple and the results of the work appear to be satisfactory.

### LICENSES FOR MASSEURS AND MANICURES.

There is a movement on foot in Albany, United States, having in view the examination of manicures, pedicures, and masseurs, before a competent authority, which shall be empowered to issue licenses renewable annually. The demand for the movement arises from the fact that at the present time there are thousands of persons in America who are practising these occupations without a vestige of qualification. In these circumstances the possession of some kind of certificate by a properly constituted body is certainly desirable.

### NOTTINGHAMSHIRE NURSING FEDERATION.

Gratifying progress in the work of the Nottinghamshire Nursing Federation was reported at the eighth annual meeting. During the year two new associations have been formed, 49,000 visits were paid to 2,745 patients, and the preliminary steps have been taken for carrying into effect the provisions of the Midwives Act. An inspector of midwives has been appointed by the Notts County Council and in the past three months she made 47 inspections. Dr. Handford expressed his confident belief, based upon experience as county medical officer, that Notts was quite in the van as regards the training of district nurses and midwives, while the chairman declared that it was impossible to overrate the use of the federation in keeping people out of the hospital. Financially the federation is in a fair position, having a balance of £166 in hand. Lady Belper, who has taken a great interest in the work from the commencement, has again been elected president.

### GAINSBOROUGH AND LEWES.

At a recent meeting of the Gainsborough Guardians it was announced that over a dozen applications had been received for the post of assistant nurse, and much gratification was expressed at the number. The experience of the Lewes Guardians is not so pleasant. The nurse who was last appointed resigned before her appointment could be confirmed, and the clerk said that the matter was becoming serious, because "the board gets so well advertised that nobody enters their service as nurse if they can go anywhere else." We are glad to learn that the General Purposes Committee are to inquire into the matter. They can achieve the same results as the Gainsborough Guardians if they go the same way to work.

### SHORT ITEMS.

Three women have been elected members of the Committee of the Royal United Hospital, Bath, and one on that of Winsley Sanatorium, Bath.

### The Nursing Outlook.

"From magnanimity, all fear above;
From nobler recompense, above applause,
Which owes to man's short outlook all its charm."

### COMMON SENSE IN NURSING AFFAIRS.

An exhibition of more common sense in the treatment of nursing questions is urgently called for at the present time. No one can have more sympathy than we have with the just aspirations of all nurses who have undertaken the work, with the single-hearted intention of learning their business and doing their best for the patient, always, in all circumstances. Everything which tends to raise the morale, to protect the interests of the patient and trained nurse, and to minister to their comfort and well-being, has and always must have, our warmest sympathy and support. We rejoice, therefore, that English nurses have displayed so much common sense and intelligence, despite all efforts to misdirect and unduly exalt them.

We should have no fault to find with those who persistently maintain that nursing is a learned profession, if they would be logical to their ideals by insisting, that no woman can be efficient as a nurse unless she possesses a medical qualification. No doubt the "stage army" will scorn this suggestion and treat it with contumely, in which case we hope they will not longer continue the attempt to mislead nurses as to the proper and well-defined limitations of a nurse's rôle, in the sick-room and in the hospital ward. A nurse, who is capable of thinking for herself, desires to be regarded as the handmaiden, not as the practitioner, of medicine. She is properly content to confine herself to her own duties, acting always under the direction of the medical attendant. The advancement of claims to a status, for instance, which has no relation to or bearing upon the true position of a trained and competent nurse, does an infinity of mischief; it can fulfil no useful purpose, except perhaps that as the number of educated nurses is increasing every day, it makes the propounders of these theories appear in the ridiculous light which best becomes their masquerading.

The chief cause of much of the present confusion is the knowledge, that a large number of women under existing circumstances pose as nurses and impose on the public as such, who have never been adequately trained, and who in fact may never have had any real training in nursing at all. The general impression abroad is, that, in order to effectually put an end to impostors of this kind, and to protect the real nurse adequately, it is essential to procure the passing of an Act of Parliament similar to the Midwives Act with penal clauses. Hence, many are inclined to advocate the State registration of nurses, believing that in this way alone can nurses who are certificated be protected as a body from such impostors. Yet, as we showed in our issue of the 14th ult., registration can never guarantee to the sick or the medical profession that they have the right nurse, that is, one not only technically capable, but also able to take her proper place in the household of her patient, where she should naturally be a comfort and a help. It cannot clear the whole nursing position, nor will it prove a panacea for the evils to which the best nurses are at present exposed. Its effects must be exceedingly limited, and for this reason, other and better means must be found to protect the really efficient nurse, and to enable the public and the profession to readily obtain her services and hers alone, when a sick attendant is required.

These means will be best supplied by a Central Council of Nursing Education, representative of all interests, which will concern itself with the higher education of nurses, and provide a minimum average standard of efficiency for all. Such an organisation, established by the co-operation of some of the more intelligent and public-spirited nurse-training schools and teachers and instructors of probationers, will create a Central Council of Nursing Education, which must have from the outset an authoritative basis. It has been feared by some that this Central Council cannot afford adequate protection to the nurses who hold its certificates against those who pretend to hold them, because it will possess no means of penalising such impostors. This is an entire misapprehension, as the Central Council of Education for Nurses will have the right under the common law to prosecute all who pretend to hold its certificate, and will take power under its constitution to use its funds for this purpose. So it will be part of its business to prosecute all women who masquerade as nurses without warrant.

It is an entire misapprehension to suppose that any special or other Act of Parliament is necessary to confer this power upon the Central Council, and in fact penal clauses in special acts in this country have proved a dead letter in the past. The common law right is ample, and the Central Nursing Council will be enabled to protect all who hold its certificates and to put down impostors effectually, for those responsible for its management will have the will, knowing the necessity, to promptly and continuously use their powers for the protection of the nurses, and also of the public. It follows that the certificate of the Central Council of Nursing Education must at once attract the larger body of working nurses, being the most efficient of their class, because holders of this Council's certificate will have the substantial advantage of being guaranteed full protection for themselves and the public, and so gradually but surely the present evils will be readily and effectually removed. We have little doubt that Parliament will recognise the wisdom of those who are responsible for the establishment of the Central Council of Nursing Education, and that it will be anxious to do all it can to extend the influence and authority of this most welcome and necessary organisation.

### Lectures upon the Nursing of Infectious Diseases.

By F. J. WOOLLACOTT, M.A., M.D., B.S.Oxon, D.P.H., Senior Assistant Medical Officer, Park Hospital, Metropolitan Asylums Board, Hither Green.

### LECTURE XIV.—SMALL-POX.

SMALL-POX or Variola is probably more intensely infectious than any other disease known in this country, and, in the absence of vaccination, is one of the most fatal. The infection is usually transmitted directly from the body of the patient, either during life or after death, but it may be readily communicated by means of clothing or other articles. It may also be conveyed for a considerable distance through the air, and it has been shown that people living in the immediate neighbourhood of small-pox hospitals are exposed to considerable risk. In consequence of this, and in order to minimise the danger as much as possible, such hospitals are no longer allowed in towns or thickly populated districts. In London, for instance, all the small-pox patients are removed several miles by river to a lonely spot near Dartford, and a similar plan has been adopted in the other large towns.

The disease in this country at the present time usually appears in the form of epidemics, which occur every few years, while in the intermediate periods it is rarely seen, and may, indeed, be entirely absent. In epidemic times it is most prevalent during the cold months of the year, and during the summer rapidly diminishes. Since Jenner's great discovery, at the end of the eighteenth century, of the value of vaccination in preventing small-pox, great changes have taken place in its prevalence and character. In previous times it was about as widespread as measles is now, there was no way of preventing it, and probably few people escaped. Great numbers died, and many more were left permanently disfigured or disabled. After the introduction of vaccination, and especially after it was made compulsory by law, the disease has steadily diminished in frequency, and there is no doubt that if vaccination and revaccination could be universally enforced it might be stamped out entirely.

The incubation period of small-pox does not vary much, and is usually 12 days from exposure to infection. At the end of that time there suddenly appear symptoms of illness, the most constant of which are headache, vomiting, and backache. The last of these is very characteristic of the disease. It is felt across the loins, and is often severe. The mouth is dry and there may be slight sore throat. The temperature rises rapidly, and rigors often occur. There is frequently more or less delirium, and in children convulsions. The patient may be restless or drowsy, and is quickly prostrated. As a rule the early symptoms are severe and the patient is obviously very ill from the first. At times, however, the symptoms, though of a similar character, are milder, and in these cases the disease usually runs a mild course throughout.

Before the characteristic eruption makes its appearance various more or less transitory rashes are occasionally seen. Many of these are erythematous in character and may resemble either scarlet fever or measles, though as a rule they are distributed somewhat unevenly on the surface of the body. Another variety, whose presence is always very suggestive of small-pox, is as follows. Small hæmorrhages, resembling fleabites and called petechiæ, appear in the skin. They are thickest on the abdomen and in the groins, but are also numerous across the loins, and may even extend to other parts of the trunk and the limbs. Their colour is a dark purple or a brick red, and the surrounding skin is often red and sometimes of a deep scarlet tint.

The eruption of small-pox begins early on the third day of the disease. It is first seen, as a rule, on the face, and soon spreads to the limbs and trunk. If very copious it may take two or three days to come out; but if scanty only a few hours. In the earliest stage it consists of small red spots, which rapidly become hard and papular, and feel like small shot embedded in the skin. Each of these papules is called a pock, and, in unvaccinated patients, undergoes the following changes. It grows steadily larger and after a day or two becomes vesicular. The vesicle increases in size and is usually fully developed on the fifth day of the eruption, when its appearance is very characteristic. It is round, translucent, and filled with a clear serum, firm to the touch, and about the size of a small pea. Its top is flat and in the centre there is usually a slight depression or dimple. In the course of the next few days its contents become cloudy, it loses its translucency and is converted into a pustule. At the same time it increases somewhat in size, swells up, and the top, instead of being flat, becomes rounded. While these changes are going on the surrounding skin becomes more and more reddened and swollen. A little later the pustule ruptures, its contents escape and dry up into a thick brownish crust or scab. In a few days this falls off and leaves a reddish brown, usually depressed, scar. At the same time the inflammation of the surrounding skin slowly subsides. In some parts of the body, especially in the thick skin of the palms and soles, the pustules do not rupture, but dry up, and then appear as small, round, brown bodies under the skin, often called "seeds." The rash is thickest on the face and limbs, especially the hands and feet, while the trunk is usually much less affected. On the abdomen particularly the pocks are rarely numerous. A curious feature of the eruption is its liking for any areas of skin which have been subjected to slight injury or pressure. Thus it is not uncommon to see the parts pressed on by the garters or the braces thickly studded with pocks, while the surrounding skin is comparatively free.

The eruption is not confined to the skin, but also involves the mucous membrane of the mouth, pharynx, and air-passages. Vesicles appear in these situations, rapidly develop, and soon rupture, leaving a dirty, ulcerated, and swollen surface, which in time slowly heals.

In patients who have been vaccinated, the eruption is very frequently of a much less severe character than that just described, assuming what is known as the *modified* form. In such cases it usually comes out rapidly, and though at times abundant, is more often scanty. The papules remain small and do not undergo the regular development of the unmodified or *natural* form of small-pox. They frequently abort without reaching the vesicular stage at all, and any vesicles that may appear are small, and often dry up without the formation of pus. There is very little swelling of the surrounding skin, and the symptoms are nearly always slight. The patient has but little discomfort, and indeed often feels perfectly well as soon as the rash has appeared. The temperature falls to normal and convalescence is uninterrupted.

As is the case in other infectious diseases, attacks of small-pox may vary very considerably in severity, and are usually classified into three varieties, according to the amount and character of the eruption—viz. (1) discrete, (2) confluent, and (3) hæmorrhagic.

(i.) *Discrete Small-pox.*—The eruption, whether modified or not, is comparatively scanty, and the pocks are for the most part separated one from another by healthy skin, and do not coalesce. The symptoms are similar to those of the confluent type, about to be described, but much less severe.

Complications are not common, and, as a rule, except in the very young, recovery follows.

(ii.) *Confluent Small pox.*—The eruption is very dense, and the pocks are so thick set that, as they develop, they coalesce one with another. In some cases so numerous are they that on the face, scalp, and limbs, where the rash is always thickest, they involve the whole of the surface, no healthy skin being left. The swelling of the skin is considerable, especially in the pustular stage, and causes so much disfigurement that patients are often not recognised by their friends. As the eruption comes out the temperature falls somewhat, but does not, as a rule, reach normal, and as the pustules develop it rises again often to 104° F. or more. This is known as the "secondary fever," and often persists for several days, gradually subsiding, in favourable cases, as the scabs fall off. The symptoms increase in intensity as the eruption advances from stage to stage. Delirium is common throughout, at first of a restless active character, but gradually becoming quieter as the patient grows weaker. The skin is hot and uncomfortable, and often intensely irritable, and the hands and feet are painful and tender. From the whole body a characteristic and unpleasant fœtor arises. The mouth and throat are sore and painful, the tongue is swollen, and saliva is constantly running away. The voice is hoarse, and more or less croup may be present. When the pustules rupture, their contents ooze out and dry up into thick crusts, which gradually separate, and the swelling of the skin subsides. At the same time, in favourable cases, the general condition improves, often very rapidly. The mind becomes clear and appetite returns. In other cases the condition grows steadily worse, the temperature remains high, delirium deepens into coma, and the patient succumbs from exhaustion, or as the result of some complication.

The swelling of the skin, which, as we have seen, is usually very great, is sometimes very little marked, owing to a peculiar form assumed by the eruption. The vesicles are less raised and flatter than usual, and, especially on the face, are so numerous and closely packed together that the surface appears more or less smooth and even, and of a dull, dirty white colour. This variety is sometimes known as the "flat" rash. It is always a sign of extreme danger and the patients rarely recover.

(iii) *Hæmorrhagic Small-pox.*—This is also known as "black" small-pox, and is almost invariably fatal. In its most characteristic form the disease runs its course very rapidly, and the patient dies before the pocks have had time to come out. The early symptoms are very severe, and the hæmorrhagic character soon shows itself. The whole skin often assumes a deep-red tint, and becomes dotted over with hæmorrhagic spots. They vary in colour from red to black, and, though at first small, soon increase in size. The most typical are the black, inky ones, which may enlarge into patches as big as the palm of the hand, or even larger. At the same time hæmorrhage is taking place into all parts of the body. Blood oozes from the mouth, nose, and vagina, and appears in the stools and urine. Very frequently there is hæmorrhage beneath the conjunctiva of the eyes. The patient, though usually quite rational, is obviously very ill, and dies in a few days.

If the characteristic small-pox rash have time to develop, the appearances are somewhat modified; but there is the same general tendency to bleeding in the skin and other parts of the body. In addition, hæmorrhages occur beneath and around the pocks.

### Infantile Mortality in Russia.

BY AN ENGLISH NURSE.

Russia stands pre-eminent amongst civilised nations for its enormous infantile death-rate. All over the country it reaches the appalling total of 35 per cent., compared with about 8 per cent. in England, and 3 per cent. in Ireland. Now the reasons for this state of things are various, but the most striking are the insanitary condition of peasant life and the gross ignorance and superstition of the people.

### A Russian Peasant Home.

Russian village life is distinctly patriarchical. The father and mother are the heads of the household, and all the sons as they marry bring their wives home into the paternal cabin and there the families are brought up. Early marriages are the rule. A lad of 17 or 18 is considered quite fit for the burden of paternity. A Russian village is an assemblage of wooden huts, each hut consisting of two rooms, usually very scantily furnished. A wooden stove occupies the centre of one room, and there is usually a small table and a couple of benches. A samovar or tea urn, some glasses for tea, crockery, and in addition a few cooking utensils, make the sum total, with the exception of pillows and a rug for sleeping. The father and mother, with as many of the children as there is room for, sleep on the top of the stove. The rest of the family simply lie with pillows under their heads on the floor in their clothes, which are taken off just once a week, on Saturday nights, when every Russian peasant enjoys the luxury of a vapour bath. I have myself seen 22 children, the offspring of four brothers, nearly all apparently under 12 years of age, living together in one cabin. Naturally, if an epidemic breaks out, the children succumb like flies in autumn. When the father and mother die, the eldest brother takes possession of the paternal abode, and the younger brothers erect wooden shanties for themselves. Russia is so under-populated and the villages so remote that in many cases a doctor comes round once a fortnight or so. The old crones, as in Ireland, practise a species of witchcraft with the object of averting the evil eye, and many of these charms are strange and weird in the extreme; but the people are exceedingly ignorant, only about 30 per cent. of the population being able to read. They are therefore particularly open to superstition.

### A Royal Wet-Nurse.

When I was in Russia there was in the Palace a wet-nurse, a married woman with a young family. One of her children had died while she was nursing another child, and she did not know of it till her return home. From what I heard I believed that the child had had no chance of life owing to the ignorance of the old granny who was to rear it. I was very sorry for the poor woman, and while she was with me I taught her all I could concerning the bringing up of children. And when she was leaving me I got the doctor to give her a little store of simple remedies—castor-oil, etc.—with plainly written instructions how and when to use them. But the next child she had after leaving the Palace died of diarrhœa after a short little life, and when I saw her again, she began to tell me of all his sufferings and what she had done to relieve them. First of all she and the granny had tried some charm, giving it about three days to act in. This failed, so her husband went off to the next village, some 20 miles away, for the doctor, who paid periodical visits to the village. He gave certain medicine and good advice concerning diet. The former was administered and the latter totally disregarded. On his next visit to the

### INFANTILE MORTALITY IN RUSSIA—*Continued.*

village the doctor came to see the little sufferer, who was then past all human aid. The child was between six and seven months' old. I asked the mother what she had given it. She was nursing it herself, but supplemented the milk with cabbage soup and finally minced mutton chop. I told her the child was much too young to take either of these, and asked her why she had not given him some castor-oil, regulated her own diet, and followed some of the directions the doctor had given her. She said that castor-oil was very suitable and good for the Imperial babies, but her child was only a peasant and used to a diet of cabbage soup, and the mutton chop was so finely minced that it really could not have harmed him. In any case had it been the will of God the child would have recovered even had nothing been done for him!

### A Poor Baby Sacrificed for a Rich One.

Another fruitful source of infantile mortality is the system of wet-nursing. I have been told that in the old slavery days this evil was far less, because it was naturally to the interest of the proprietor to guard infant life. But at present every mother who can possibly afford it, takes a peasant woman to bring up her child. One of the reasons for this is the idea that a young country woman is better adapted to feed the child than the infant's mother, whose amusements would interfere with the regularity of its meals. In 99 per cent. of such cases, the little peasant child dies a slow, lingering death of starvation, for in the villages no one understands how to bring up a baby by hand. Roughly speaking, therefore, for every child born into the better classes a peasant baby is sacrificed, and during my stay in Russia, nothing distressed me more than the utter callousness displayed by the rich to these perfectly preventable sorrows of the poor, and their indifference to the manifest harm done to the country in general by the loss of these little ones. But there is no public spirit in Russia. The better classes seem to forget that the same God made both peasant and peer, that each has an equally God-given right to live, and that the tears of the peasant are just as precious in His sight as those of the most highly born in the land. However, I never could get any Russian to agree with me, and was generally told that better-class women suffer more for their children than do the humbler women, whose sufferings are not to be weighed in the balance with those of their wealthier sisters. Just now it is becoming the fashion to put the child of the wet-nurse into an institution with another woman to nurse and look after it. I asked a lady, who told me with great pride that her wet-nurse's baby was being thus provided for, what became of the child of the other woman, and she was quite amazed at the question. "How does that concern me?" she asked in genuine surprise. "Probably she did not care for the child, and was glad to get rid of it."

### Russian Objection to Feeding-Bottles.

If you speak to Russian women of the lower class of the virtues of a nice clean feeding-bottle for a child whose mother is unable to nurse it, they look at you in pained surprise, and tell you patiently and gently that human milk is the only natural diet for a child. They then add that you don't understand. As I am unmarried, I was often told that I did not and could not understand the feelings of a mother. A mother, they said, knew by instinct how to treat her child. Studying the subject was no use at all. Whatever the best nurses and doctors in the world knew was as nothing compared to a mother's knowledge! A large proportion of children in Russia are illegitimate, and consequently there are numbers of foundling institutes throughout Russia. During my career there I met several of these foundlings, and worse brought up young women it never was my lot to come across. The Russian Government gives all such unfortunate mothers a pension of nine roubles a month—about £1 of our money—if they will stay at home and look after their children for a year. Many of them do it, but others are tempted by the higher wages, the beautiful costumes, and the luxurious home offered to wet-nurses, and give their children over to the care of some ignorant old village woman to bring up, agreeing that for a year they will endeavour to hold no communication with, nor receive any news of, their own little ones. Of what happens when, with the year's wages in their pocket, the fine dresses on their back, laden with presents, praises, and thanks ringing in their ears, they return to find the abandoned child has died of starvation, I shall not speak. The fact that it is almost impossible to get a woman to act as wet-nurse a second time shows pretty clearly that all their gains are but as dead sea-fruit.

### How Wet-Nurses are Procured.

In all big towns there are institutes from which wet nurses are hired. They have generally speaking a maternity hospital attached. Here the poor women are received and kindly tended in their illness, and about a fortnight after the birth of their own child they are sent out. It was my fortune to meet with the head of one of these institutes. She told me that she had visited last year over 200 villages searching for likely young women. Matters must be arranged with herself, her husband, and his father and mother. The emissary from the institute is naturally skilled in argument. She points out the very material advantages to be gained, tells the woman that with her year's wages she can build a home for herself, she flatters the grandmother, scoffs at the idea of the expected little one being in danger of privation, and says that the child is in the hands of God. She generally carries her point, and so the Massacre of the Innocents goes on. When a birth is expected in the Imperial family this is held out as a great inducement, and it is hinted that there is no knowing whether the woman who is now wavering may not be the one chosen to bring up the future Emperor of Russia. After this suggestion, the added assurance that in such an event the wet-nurse's fortune would be made, and that she would be rich for life, is hardly needed. The idea of being able to render such a service to the Tsar, whom they call "the God upon Earth," is generally sufficient to settle the bargain.

### Presentations.

Rainhill County Asylum.—Miss Valentine, who is resigning the post of matron at the main building, County Asylum, Rainhill, has been presented by the nursing staff with a handsome silver fitted dressing case. Owing to ill-health, she intends to take a long and well-earned rest.

Tonbridge Union Infirmary.—Upon leaving to take up her new duties at Farnham Infirmary, Miss Tisdell, superintendent nurse of Tonbridge Union Infirmary, was presented with a handsome silver travelling clock from the medical and nursing staff, as a token of their appreciation of her work during the five years she was among them.

Merton District Nursing Association.—The Vicar of Merton, Surrey, has presented Nurse Louise White, on behalf of the nursing committee and parishioners, with a travelling case, a jewel case, and cruet-stand as a token of appreciation of her services during the past three years. She has also been the recipient of numerous useful gifts from her patients. She is leaving to take up private nursing.

### Appointments.

[No charge is made for announcements under this head, and we are always glad to receive, and publish, appointments. The information, to insure accuracy, should be sent from the nurses themselves, and we cannot undertake to correct official announcements which may happen to be inaccurate. It is essential that in all cases the school of training should be given.]

BRIGHTON WORKHOUSE INFIRMARY.—Miss Annie Moase has been appointed charge nurse of a male block. Miss Florence Lewis charge nurse of the Children's Ward, and Miss Nellie Whitney staff nurse. Miss Moase was trained at St. Olave's Infirmary, and was subsequently private nurse at Bradford, charge nurse at Brighton Infirmary, of the Children's Ward, and afterwards of a Women's Ward. Miss Lewis and Miss Whitney were both trained at Brighton Workhouse Infirmary, and both hold the L.O.S. certificate.

BROMPTON HOSPITAL SANATORIUM AND CONVALESCENT HOME, FRIMLEY.—Miss Irene Webb has been appointed matron. She was trained at the London Hospital. She has since been head nurse at the Royal Hospital, Putney; charge nurse at the Western Hospital, Fulham; night superintendent at the Grove Hospital, Tooting; assistant matron at the Fountain Hospital, Tooting; and matron of the Borough Isolation Hospital, Leicester.

EASTVILLE WORKHOUSE, BRISTOL.—Miss Clara Brown and Miss Annie Grice have been appointed charge nurses. Miss Brown was trained at Steyning Union Infirmary and has since been charge nurse at Colchester Union Infirmary. Miss Grice was trained at Allt-yr-yn Hospital, Newport, Monmouthshire. She holds the L.O.S. certificate.

EPSOM ISOLATION HOSPITAL.—Miss Agnes Corrie has been appointed nurse-matron. She was trained at North Staffordshire Infirmary, Stoke-on-Trent, and has since been district nurse in Bermondsey and North Wales, charge nurse at the Eastern Fever Hospital, London, and sister at Southwark Infirmary. She has also done private nursing.

GLASGOW LOCK HOSPITAL.—Miss Eliza Leah has been appointed charge nurse. She was trained at Bradford Union Hospital, and has since been nurse at the Manchester Lock, and Royal Portsmouth, Hospitals.

KINGSTON-UPON-HULL SANATORIUM.—Miss Katrine Ediss has been appointed sister. She was trained at the Western Infirmary, Glasgow, and has since been nurse at Belvidere Fever Hospital, Glasgow, and Little Bromwich Hospital, Birmingham.

MANCHESTER CONSUMPTION HOSPITAL, BOWDON.—Miss K. H. Wheatley has been appointed charge nurse. She was trained at Lambeth Infirmary, and has since been charge nurse in the same institution, sister at Rotherham Hospital, sister at The Retreat, York, and sister at the Royal Chest Hospital, City Road, London.

NORTH MIDLAND INEBRIATES REFORMATORY, ACKWORTH, YORKSHIRE.—Miss Emily L. Callaway has been appointed assistant matron. She was trained at the Workhouse Infirmary, Walsall, and has since been private nurse, midwife at the Brighton Workhouse Infirmary, and charge nurse of a male block in the same institution. She holds the L.O.S. certificate.

ROCHFORD UNION INFIRMARY.—Miss Ellen Hinson has been appointed charge nurse. She was trained at St. George's-in-the-East Infirmary. She has since been staff nurse at the same institution.

ROYAL WESTMINSTER OPHTHALMIC HOSPITAL. — Miss Mary Connell has been appointed sister. She was trained at the Sheffield Royal Infirmary; she has since been sister at the Burton-on-Trent Infirmary, and theatre and ward sister at the Liverpool Stanley Hospital.

TONBRIDGE INFIRMARY.—Miss E. A. Robb has been appointed superintendent nurse, and Miss Ellen Farman charge nurse. Miss Robb was trained at Mile End Infirmary, London, where she was afterwards staff nurse. She has since been superintendent nurse at Whitechapel Workhouse, superintendent nurse at Alcester Infirmary, and senior charge nurse at Tonbridge Infirmary. She holds the L.O.S. and the Central Midwives Board certificates. Miss Farman was trained at Tonbridge Infirmary, and has since been staff nurse in the same institution.

UNION HOSPITAL, BRADFORD.—Miss Florence E. M. Gibbs has been appointed sister. She was trained at Kingston-on-Thames Union Infirmary and St. Peter's Home, Kilburn, and she has been nurse at Manchester Ear Hospital.

### Everybody's Opinion.

### THE DUTIES OF A DISTRICT MIDWIFE.

"A CONSTANT READER" writes: I was glad to see the letter from "W." on the appointments of inspectors of midwives. I should like especially to emphasise the matter of inadequate pay and the trying circumstances connected with the duties of a district midwife. For instance, most cases of midwifery are night work, and call for a great deal of energy. The midwife has to walk many miles before and after the event, and she requires more meals, thereby increasing her expenditure. The Central Midwives Board desires her to wear a washable material, this, too, takes a good portion of her small income. It should also be considered that she has to go at any moment, night or day. When these matters are considered your readers must, I think, agree that every encouragement should be given to midwives by promotion, etc., or otherwise we cannot expect women of energy and education to enter our ranks.

### ANOTHER WARNING FROM SOUTH AFRICA.

"SISTER MARY" writes from Johannesburg, under date of January 16th, 1905: I was indeed very pleased to see a letter in THE HOSPITAL of December 10th headed "Another Warning from South Africa." If nurses would only heed these warnings how much better it would be for themselves and those nurses who are already here; we are not selfish and would gladly welcome more nurses here if it were possible for us all to make a living. A doctor told me the other day that there are about 800 nurses in Johannesburg, which means about one nurse to every hundred of the population. In addition to this more than half of this hundred cannot afford to employ a nurse if they have sickness in the house, and a greater number of the remaining half prefer to have untrained women, not that their fees are lower—they charge exactly the same as a trained nurse—but they are more willing to act as general servants in addition to nursing — this suits the middle-class Joburger. I know a fully-trained nurse with ever so many certificates who during the last two months, after she had paid board and percentage, had 15s. left. There is not a Kaffir here who would work for 7s. 6d. a month. Some may say, "why not take hospital appointments?" Here again, any position that is worth having is not to be got, unless you have some "power behind the throne." In the general hospital a sister's post is worth £6 a month. An educated woman who has given several years of her life to qualify for this position, and they offer her £6 a month! I was nursing not long ago in a house where they had a coloured girl and kept a boy to do the dirty work for her, two in family, and she got £6 10s. a month; a good white cook gets £10, and will leave you at a moment's notice if you vex her. Nurses here are indeed badly paid, when one remembers how expensive everything is, and I can only endorse Sister Francis' warning. Would that some abler pen than mine would take the matter up and advise nurses not to come to South Africa. In fact, we are manufacturing an article in the local hospitals which we label "certificated nurse," and the supply exceeds the demand.

### Echoes from the Outside World.

### Movements of Royalty.

On Saturday afternoon the King left Buckingham Palace on a visit to Lord Rosebery, at Mentmore, Leighton Buzzard, travelling by motor-car. On Monday afternoon his Majesty paid a visit to Lord Rothschild, at Tring Park, and on Tuesday to Stowe House, where Lady Kinloss has resided since the death of the Comte de Paris.—His Majesty inspected the State rooms, and was much interested in the elephant trappings given by him as Prince of Wales, during his tour in India, to the late Duke of Buckingham, at that time Governor of Madras.—The Prince of Wales left Dublin Castle for England on Saturday morning, driving in an open carriage with the Lord Lieutenant to the railway station. His visit to Ireland was of a private character, but both in Connaught, where he had very good sport, and in Dublin the Prince moved freely and quietly among the people, and was received everywhere with the most cordial courtesy.

### The Condition of Russia.

Count Lamsdorff has formally disavowed the action of the Prefect of Police at Moscow in placarding charges against Great Britain of having fomented the strikes. The prefect has been censured, and he has been ordered to remove the placards. Last week the Tsar received a deputation of workmen of St. Petersburg at Tsarskoe Selo, and many misleading statements were made in conseqaence. There is no doubt, however, that his Majesty told the workmen that he would take measures which would secure an improvement in their lot. On Tuesday it was announced that the St. Petersburg workmen have resolved to ask the Tsar to receive a deputation chosen by themselves; the deputation he has already seen, they allege, having been selected by the employers, was not representative. The nobles of St. Petersburg have decided to present an address tc his Majesty urging the maintenance of the union between an "autocratic monarch and a devoted nation" by the admission to the councils of the State of elected representatives.

### The War in the Far East.

The first shot in the war between Russia and Japan was fired a year ago this week, and peace does not appear to be yet within measurable distance. According to the latest official reports the Russians are again showing some signs of activity on the Japanese left, but all their attacks have been repulsed. There is much talk about the return of General Kuropatkin, who is reported to be suffering from cerebral anæmia. General Stössel has publicly denied that the surrender of Port Arthur was not justified.

### A Woman Inspector under the Education Board.

A new departure has been made by the Board of Education, and the Hon. Maude Lawrence has been appointed chief woman inspector. Miss Lawrence is the youngest daughter of the first Lord Lawrence, Governor-General of India, who was chairman of the first School Board for London. She was for many years chairman of a committee of managers under the London School Board, and was a member of the London School Board itself from 1899 to 1904. In the latter year she was placed by the London County Council upon their Education Committee. Her duties will include direction of a staff of women inspectors of special qualifications and varied experience, who will assist the Board in dealing with many questions hitherto not treated sufficiently fully. Amongst these are the special education of girls and young women in public elementary schools, secondary schools, evening schools, and training colleges, and the suitability of the arrangements made for the boarding of women students in training colleges and hostels. The Board desire to direct the attention of local authorities to everything which bears upon the national physique as affected by the studies, the life, and the treatment of very young children, and of the need for providing as part of the educational system ample opportunities for girls to obtain a training for home life, simple, practical, and adapted. All these points they consider will be most efficiently looked after by a competent staff of women inspectors.

### Women as Architects.

On Monday evening the President of the Royal Institute of British Architects congratulated Miss Ethel Charles, of Flushing, near Falmouth, upon her success in carrying off the Institute Silver Medal and 20 guineas for an essay on the Development of Architectural Art from Structural Requirements and Nature of Materials. The award was gained in competition with many male students. Miss Charles is the first Lady Associate of the Institute, and was admitted three years ago. The only other lady who has been admitted so far is her sister.

### Collapse of the Tea Pension Scheme.

On Tuesday an order was granted by Mr. Justice Buckley, in the Chancery Division, for the compulsory winding-up of Nelson and Company, the proceedings having originated in connection with their tea pension scheme for widows which, it appeared, the funds of the company were insufficient to carry out. The pension, it was stated, multiplied till the profits disappeared, and payment could not be made to the 19,000 widows on the company's list.

### Destruction of More Historic Homes.

Great Gaddesden Place, Hemel Hempstead, which has for centuries been the home of the Halsey family, but which had been temporarily occupied by Mr. John Kerr, M.P., was totally destroyed by fire last week. Mrs. Kerr lost all her dresses, valuables, and jewellery, and Mrs. Baird, the lately married daughter, her entire trousseau, although, fortunately, her wedding presents were saved. Mr. Kerr rendered great assistance in his pyjamas, dressing-gown, top coat, and slippers. All the inmates escaped without injury, but in the evening after the fire, when the butler and footman were engaged in the cellar, one of the walls gave way and they were both killed.—On Friday night The Glen, the historical mansion belonging to Sir Charles Tennant, in Peebleshire, was almost entirely destroyed by fire, the west wing alone escaping. The fire broke out in a servant's bedroom, and the fire brigade on the establishment proved unable to cope with the outbreak. Some valuable works were saved, and no life was lost. Sir Charles Tennant bought The Glen for £35,000, and has spent £50,000 in improvements.

### American Evangelists in London.

On Saturday Dr. Torrey and Mr. Charles M. Alexander, two American evangelists, who have for several years been carrying on missions with great success in Australia and in the leading provincial towns of the United Kingdom, opened their campaign in London. Dr. Torrey is the pastor of a church in Chicago, and considerably the senior of Mr. Alexander, whose wife is a daughter of the late Mr. Richard Cadbury. The meetings are held in the Albert Hall at half-past three and eight, except on Sundays, when there is only one meeting, and on Mondays when in future no meeting will take place. All seats are free. The two great features of the service are the singing of Mr. Alexander, who leads a choir of over 1,000 voices, and the addresses of Dr. Torrey.

### A Book and its Story.

### A YORKSHIRE IDYLL.*

WHETHER the story of the postman poet, which Mrs. Fred. Reynolds tells in her "Book of Angelus Drayton," is taken from life or not, and the extract from the *Daily Telegraph* at the end of the diary suggests that it has its foundation in fact, it is one that will appeal to those readers who appreciate a simple story told with quiet directness, and having an undercurrent of deep pathos which adds to its charm.

The calendar of the year, from January to December, runs through the book. Not an entry for every day, but just fugitive jottings, notes on nature, and things and persons that the "gentleman postman" meets on his rural rounds. The first chapter of the book is autobiographical, and contains some of the best passages. There is distinct charm in the descriptions of the home life, the daily doings on the farm, and the delineation of the characters of Angelus Drayton's people. Later, when the record passes from facts to a reiterated chronicle of impressions made on the imaginative soul of the writer, by the change of the seasons, the rise or fall of the barometer, or by the presence of the lady to whom he plays the part of devout lover, the book as a whole is weakened. We have to believe that behind these revelations is the heroic personality of a man handicapped from the outset by unusual drawbacks. Yet, in spite of the sympathy which the idea arouses, the feeling remains that had the book been the work of the man himself, so far as this part of it is concerned, he would have been a little less liberal with the use of emotional elements as a stimulus to expression. But, then, the writer is not a man; and many readers of "Angelus Drayton's Book" may not, if they are women, find cause for complaint on this score. In passing, the happy selection of title headings to the chapters must be noticed, each of which is suggestive of a poem in itself. For instance, among others, "The first gift of the year," "Spring calls to the year," "The Holy day of the year," "The storm time of the year," "Love lays his finger on the year," and so on, to the ending of the book of Angelus Drayton. He speaks of himself and the circumstances that have combined to place him in his present position when the story opens as follows: "A country postman and no chance of anything better! It seems but a sorry end to all my hopes and ambitions; yet believe me it has its compensations. I am bound, indeed, to but two routes; up the winding lanes to the crest of the hill, down the long dip to Ribble, returning by the Fells, every morning; and away to Ellerdale, back through the woods and along the river bank, every afternoon. Yet God in His goodness knows there will be no sameness; the book of His writing, the pictures of His Divine fashioning, are ever new, ever changing—storm, snow, wind and shine, starlight or mist, ever varied, ever beautiful. The year lies all before me with its flowers yet unborn, its songs yet unsung; and if I set myself to tell the story of my "Wander Year," it is not because I have great gifts of writing—I would indeed it were so. I am but a plain man, and can but set things down plainly as I see them. . . . In any case my diary will fill the winter evenings which are long, whilst my books are few and oft read. My mother, too, God bless her, grows deafer as years increase upon her, and spends long hours dozing before the fire in her rocking-chair, with its cushions of faded red. . . . My mother is only a peasant, a daughter of the people, and the very best woman that God ever gave to us sons of men." After a graphic description of the interior of the house-room of the old farm-house where Angelus Drayton and his widowed mother spend their time, when his journeys are finished for the day, he then speaks again: "As for myself, I sit somewhat in the shadow, the place I hope to keep throughout these pages; near me burns a reading-lamp, relic of other days. It is with my left hand I write these words; my right sleeve is pinned to my chest empty; my right leg is stretched straight and stiff before me. I cannot bend the knee. . . . Such a very ordinary tale, yet filled with a certain tragedy—at any rate, for me. A young and restive horse, too sharp a turn at the bridge corner; then blank unconsciousness, followed by weary weeks in the Leeds Infirmary. I returned home at last minus the right arm, and with the right knee so stiffened at the joint that, though my powers as a walker are practically unimpaired, I shall always be lame." The story of Angelus Drayton is a sad one, and yet, as told, his hidden sources of joy outbalanced those of many happier lives, viewed objectively. He came of a mixed parentage socially. On his mother's side the family had for some generations been well-to-do farmers. His father was a "scholar and a gentleman" who, when a young man, had been sent by a neighbouring squire to the farm to recruit after an illness. This was followed by a relapse through which the farmer's daughter nursed him. Angelus, in speaking of the past and the first meeting of his parents says: "He came seeking health. Our people had never taken lodgers before but gave him welcome to oblige the squire who was in some way interested in him. I do not know quite how it all came about, for mother seldom speaks of him; but she told me once that he was a gentleman born and bred and that she was never worthy of him. That I did not believe nor ever shall, for there is no man that ever stepped but would be made richer and better by my mother's love. . . . He was younger than my mother by nearly twenty years, for she was past her first youth at that time and he was only thirty—just thirty by the gravestone in yonder churchyard—when he died. Looking back sometimes into the misty past it seems almost as though my mother were his mother too. I know she looked after us both as we played together, he and I . . . and both alike we loved her. I have heard she nursed him through a long illness, for he fell worse soon after he came among us, and when he got better they were married at the little church. My father was a born poet, if ever there was one; things touched by light word of his received new life, became other, more wondrous than they were before. I have—the most precious of all my possessions—a ltttle volume of his poems, bound in thin leather, quaint and old. Very beautiful and dainty are the verses therein, still, not as fine, it seems to me, as those he used to make for me on the open hill-side. The date of the book is two years before my father's marriage, and it is dedicated to one Sibyl. My mother's name, I think I have said, is Mary. But I like to think her life grew fuller and sweeter by the love of the thin, pale-faced man, with the wonderful dark eyes and the voice which sounded so different from the rough, country-side talk of the rest." Angelus was seven years old when he lost his father. He had inherited his abilities, and his ambition was to become "a gentleman and a scholar" also. How his ambitions were frustrated, how he willingly sacrificed his futuie to the interests of others our readers can see in his book to which we cordially commend them.

* The Book of Angelus Drayton. By Mrs. F. Reynolds. John Long. 6s.

### Notes and Queries.

**REGULATIONS.**

**The Editor is always willing to answer in this column, without any fee, all reasonable questions, as soon as possible.**

**But the following rules must be carefully observed :—**

**1. Every communication must be accompanied by the name and address of the writer.**

**2. The question must always bear upon nursing, directly or indirectly.**

**If an answer is required by letter a fee of half-a-crown must be enclosed with the note containing the inquiry.**

*Male Nurses and the Pension Fund.*

(140) Can a male nurse join the Royal National Pension Fund? —*H. H. W.*

Certainly, on the same conditions as a female nurse.

*Midwifery Training School.*

(141) Can you kindly let me know if there is a recognised training school for midwifery in Yorkshire or Lancashire?—*A. W.*

The Leeds Union Infirmary and Training School, the Liverpool Mill Road Infirmary, the Manchester Maternity Hospital, and the Manchester and Salford Lying-in Hospital are all recognised training schools.

*Training.*

(142) I am anxious to enter the nursing profession but have no idea how to set about it. Will you kindly inform me how I can obtain particulars. I thought it probable that I could procure a guide, or some work to that effect, which would instruct me as to the best way of securing a nurse's position in a hospital, and the necessary requirements.—*J. J.*

See "The Nursing Profession: How and Where to Train," which will give you full information.

*Homes and Hospitals.*

(143) Can you tell me of any home or hospital for paralysed children for a boy of 10 or 11 who is able to walk but can only use his right arm a little? Somewhere in the North preferred.—*L. M. T.*

There are no hospitals or homes specially for paralysis in the North of England, but write to the secretary of the Liverpool Country Hospital for Children, where the boy might possibly be eligible for admission.

(144) 1. I am anxious to hear of a home where a girl suffering from epileptic fits can be taken in. Her parents are willing to pay for her. 2. Also, I wish to hear of a home or hospital for a man suffering from cancer of the mouth. He is quite incurable, and I am afraid no payment could be made for him.—*Queen's Nurse.*

1. Write to the Hon. Sec. of the Home for Epileptics, Maghull, Liverpool, if you wish for a home in the North of England, otherwise you might apply to the Meath Home of Comfort for Female Epileptics, Westbrook, Godalming; also to St. Luke's Home for Epileptic Churchwomen, 36 Parkwood Road, Bournemouth. 2. Write to the Secretary of the Liverpool Hospital for Cancer and Skin Diseases; also to the Secretary of the Christie Cancer Hospital, Oxford Street, Manchester. Then the London Cancer Hospital, Fulham Road, S.W., has a number of (free) beds for the use of patients, who may remain for life.

*Queen Alexandra's Nursing Home.*

(145) Can you give me any information as to how I could enter the Queen Alexandra's Nursing Home?—*J. H. O'B.*

There is no such home. Do you mean Queen Alexandra's Imperial Military Nursing Service? If so, write for particulars to the Under-Secretary of State, War Office, Pall Mall, S.W.

*Gynæcology.*

(146) Can a certificate be taken for gynæcological nursing after three or six months' special training, and where can such training be obtained in London?—*Alpha.*

Write to the Secretary of the British Gynæcological Society, 437 Oxford Street, W.

**Handbooks for Nurses.**

| | Post Free. |
|---|---|
| How to Become a Nurse: "The Nursing Profession" ... | 2s. 4d. |
| "Nurses' Pronouncing Dictionary." Terms and Treatment... ... ... ... ... ... ... ... | 2s. 0d. |
| "A Handbook for Nurses." Dr J. K. Watson ... ... | 5s. 4d. |
| "Elementary Physiology for Nurses" ... ... ... | 2s. 0d. |
| "Swedish Massage and Movements" ... ... ... | 2s. 6d. |

Of all booksellers or of The Scientific Press, Limited, 28 & 29 Southampton Street, Strand, London, W.C.

### For Reading to the Sick.

THE SYMPATHY OF CHRIST.

As oft with worn and weary feet
We tread earth's rugged valley o'er,
The thought, how comforting, how sweet!—
Christ trod this toilsome path before;
Our wants and weaknesses He knows
From life's first dawning to its close.

Do sickness, feebleness, or pain
Or sorrow in our path appear?
The sweet remembrance will remain—
More deeply did He suffer here.
His life, how truly sad and brief,
Filled up with sorrow, pain, and grief.

Just such as I, this earth He trod,
With every human ill but sin;
And though, indeed, the very God,
As I am now, so He has been.
My God, my saviour, look on me
With pity, love, and sympathy!

*Wilberforce.*

It is often supposed that the spirit of holy poverty has no universal application, but is confined to those who are actually poor, or have become so by one of the vows of the religious life.

Now this spirit of poverty has a special application to the sick. The sick and suffering are always (let us hope) objects of care to those around them. They are thus enriched with comforts, sympathy, and attention—in a word, with all that money can buy and kind hearts can supply. Let them be grateful for all this, and see in it their heavenly Father's solicitude for their welfare. They must not imagine it to be a part of the spirit of holy poverty not to ask for medicines, food, and other remedies when they need them; not to remind their attendants of the doctor's orders, should they have slipped their memory. They must speak of these things, even as they would ask for them and supply them to another on whom they were in attendance. No, the spirit of poverty does not lie in that direction.

But let it be remembered that sickness in general is poverty; it means the privation of certain pleasures of life, which others enjoy; it means using one room instead of twenty; it means seeing one friend instead of fifty; it means to have no sight of the beauties and sounds of earth. That is holy poverty, when these very real privations are accepted generously, with holy joy, without a repining word, and are borne for the love of the Master. . . . The blind fiddler earning coppers by the roadside, the poor patient on a bed of pain; these may be leading lives of continued and beautiful prayer, as truly as the cloistered religious who remains on bended knee for hours day by day. Believe this, and act upon it; take it deeply to heart at this time; let it console and encourage you. *Qui laborat orat*—"he that works also prays"—is an old and true maxim. "He that suffers with patience and resignation prays continually and well."—*Anon.*

So tired: Lord, Thou wilt come
To take me to my Home,
So long desired.
Only Thy grace and mercy send,
That I may serve Thee to the end,
Though I am tired.

*M. E. Townsend.*

